# Prefrontal Hierarchical Control of Corticomuscular Coherence in Stroke

**DOI:** 10.1109/TNSRE.2026.3685567

**Published:** 2026

**Authors:** Jasper I. Mark, Rachana Gangwani, Justin Riddle, Jessica M. Cassidy

**Affiliations:** Department of Health Sciences, The University of North Carolina at Chapel Hill, Chapel Hill, NC 27514 USA; Jefferson Moss Rehabilitation Research Institute, Elkins Park, PA 19027 USA; Department of Psychology, Florida State University, Tallahassee, FL 32306 USA; Department of Health Sciences, The University of North Carolina at Chapel Hill, Chapel Hill, NC 27514 USA

**Keywords:** Cross-frequency coupling, EEG, EMG, motor control, neurorehabilitation, signal processing

## Abstract

Stroke recovery involves reorganization of large-scale brain networks, yet most neurophysiological biomarkers assess either motor or cognitive function in isolation. To address this limitation, we introduce phase-modulated corticomuscular coherence (pmCMC), a novel metric that quantifies how prefrontal delta-phase activity modulates beta-band corticomuscular coherence between the primary motor cortex and peripheral muscle. This approach integrates cross-frequency and corticomuscular coupling, offering a dynamic index of cognitive-motor interaction. We applied pmCMC to a cohort of individuals with hemiparetic stroke who performed a precision grip task during high-density EEG and EMG recording at admission and discharge from an inpatient rehabilitation facility. Our findings reveal that pmCMC increases across rehabilitation and positively relates to corticostriatal but not corticospinal tract injury. Additionally, higher pmCMC at admission was positively associated with greater gains in motor coordination, functional independence, and cognitive status. These results position pmCMC as hybrid neurophysiological measure of cognitive-motor integration relevant to post-stroke recovery and encourages further investigation of its prognostic utility, causal mechanisms, and applicability across diverse motor tasks and clinical populations.

## I. Introduction

Stroke recovery is not confined to isolated circuits, but instead reflects the reorganization of large-scale brain networks spanning cognitive, sensorimotor, and subcortical systems [1], [2], [3]. Such network-level reorganization implies that recovery-related changes are distributed across interacting systems rather than localized to a single region or pathway. Following stroke, the brain engages compensatory mechanisms that involve not only the primary motor cortex (M1) but also prefrontal, parietal, and subcortical regions responsible for planning, attention, and cognitive control [1], [4], [5], [6]. This distributed plasticity reflects the brain’s attempt to scaffold impaired sensorimotor pathways with higher-order executive systems, particularly in individuals with more severe impairment [6], [7], [8]. However, current electrophysiological biomarkers of recovery typically focus on motor or cognitive domain-specific measures separately [9], such as bilateral M1 coherence [10], [11] or prefrontal activity [12], [13], respectively. Approaches that capture cognitive and motor system interactions may enhance our mechanistic understanding of stroke recovery by affording a more accurate portrayal of post-stroke network reorganization.

Corticomuscular coherence (CMC) is a well-established motor-related biomarker [14] that quantifies the synchronization of oscillatory activity between the motor cortex and muscles acquired from electroencephalography (EEG), and electromyography (EMG), respectively [15]. As it conveys coupling between M1 and spinal motor units, CMC, particularly in the beta frequency range (13-30 Hz) during isometric tasks, represents descending motor pathway contributions rather than the influence of higher-order cognitive control processes [16], [17]. Following stroke, motor task performance often requires greater involvement of executive systems to initiate and sustain movement [18], [19], [20], [21]. This reveals a shortcoming of CMC measurement to capture the increased cognitive contribution to motor execution in the post-stroke brain.

Additionally, prior work shows that in individuals with severe motor impairment, CMC becomes spatially diffuse, with coherence extending beyond contralateral M1 into both ipsilesional and contralesional cortical regions [22], [23]. Several studies have also reported anterior shifts in CMC away from ipsilesional M1 (iM1) [24], [25]. The functional significance of these anterior shifts remains unclear, with some interpreting them as compensatory recruitment of secondary motor regions and others as maladaptive reorganization indicative of inefficient motor control strategies. Collectively, these findings underscore the utility of CMC as an informative marker of corticospinal communication but also highlight uncertainty regarding whether beta-band CMC solely reflects descending motor output or whether it is influenced by higher-order processes such as executive control. Moreover, CMC does not reveal how descending motor output is shaped by cognitive control factors such as attention, executive monitoring, or task regulation.

To address these limitations of CMC and to better model the integration of cognitive and motor system function, recent work has examined *cross-frequency coupling*. This electrophysiological approach captures interregional coordination across different frequency bands [26], [27]. Phase-amplitude coupling (PAC), a type of cross-frequency coupling, quantifies how the phase of a low-frequency oscillatory activity modulates the amplitude of a higher-frequency activity [28], [29]. This phenomenon is thought to exemplify hierarchical control, whereby slower oscillations in higher-order regions temporally organize faster sensorimotor processes across distributed circuits [29].

Studies of PAC were initially conducted in the cognitive neurosciences [26], [30], [31], but recent investigations have expanded clinically to include cohorts with neuromuscular disorders [28], [32], including stroke [6], [33] and Parkinson’s disease [34]. Of particular relevance is delta-band activity (1–4 Hz) in the prefrontal cortex (PFC), which is implicated in attention, executive function, and volitional motor control [29], [35]. Prior work has shown that delta–beta PAC between PFC and M1 supports top-down modulation during goal-directed force tasks [36]. Notably, this coupling was elevated early following stroke and was associated with better functional motor status and greater recovery [6]. These findings suggest that top-down influence from PFC over M1, conveyed through low-frequency phase modulation, indicates the recruitment of compensatory strategies to mitigate motor deficits [6], [37]. Understanding how coordination between PFC and M1 translates into effective motor output at the level of muscle activation is an important next step. Examining the anatomical pathways and their relationship with neural oscillatory dynamics, as captured by CMC and PAC, is therefore essential.

The corticospinal tract (CST) provides the primary conduit for motor output from M1 to the periphery [38], serving as the structural substrate of CMC [15], [39]. In parallel, corticostriatal pathways link prefrontal regions with the basal ganglia and M1 [40], forming closed-loop circuits that facilitate cognitive control over action selection, timing, and goal-directed movement [41], [42], [43]. These corticostriatal circuits are well-positioned to modulate prefrontomotor PAC and downstream motor signaling [44], [45], [46], [47]. Together, CST and corticostriatal pathways shape top-down and bottom-up interactions within the motor system and may govern the capacity for cognitive scaffolding in the face of sensorimotor disruption [43], [48]. Despite the insight into cognitive and motor system function provided by PAC and CMC–as supported by their structural correlates–a critical gap remains: the absence of a neurophysiological measure that links cognitive control signals to motor output in a temporally resolved and mechanistically interpretable manner.

Building on these insights, we introduce phase-modulated corticomuscular coherence (pmCMC), a novel biomarker designed to unify the hierarchical influence of PAC with the directional sensorimotor coupling captured by CMC. Rather than treating PAC and CMC as separate phenomena, pmCMC quantifies the degree to which CMC is dynamically modulated by slow-phase activity from higher-order regions such as the PFC. Unlike conventional PAC measurement, which quantifies interactions between neural signals, pmCMC assesses the degree to which low-frequency prefrontal phase influences CMC, thereby indexing hierarchical control over descending motor pathways. This approach allows us to examine the *cognitive* regulation of descending motor pathways that may signify the compensatory recruitment of prefrontal control signals.

We applied this conceptual framework to early stroke recovery. Individuals with hemiparetic stroke completed a precision isometric grip task at two time points: around the time of admission to and discharge from an inpatient rehabilitation facility (IRF). We hypothesized that prefrontal delta phase activity would influence beta CMC between iM1 and the affected first dorsal interosseous (FDI) muscle during the grip task, capturing the increased top-down scaffolding required to compensate for impaired motor control. Given that motor tasks may impose increased cognitive demand on individuals post-stroke [21], we hypothesized that pmCMC tracks with symptom expression such that it would be elevated around the time of IRF admission, consistent with compensatory upregulation of cognitive control over sensorimotor pathways, and would decrease over the course of inpatient rehabilitation in parallel with functional improvements.

## II. Methodology

### A. Participants

The present study was a prospective observational study approved by the Institutional Review Board of the University of North Carolina at Chapel Hill. All participants provided written informed consent prior to enrollment. The study was conducted between November 2020 and February 2022. Individuals aged ≥ 18 years with a radiologically confirmed ischemic or hemorrhagic stroke were recruited during their IRF stay. Participants completed two study visits near the time of IRF admission and discharge. Visits involved task-based EEG/EMG recordings and behavioral assessments to evaluate motor impairment and cognition.

### B. Electrophysiology Acquisition and Processing

High-density EEG and EMG data were collected with a 256-channel Hydrocel Geodesic Sensor Net (EGI, Eugene, OR) with signals sampled at 1000 Hz. During EEG/EMG recordings, participants engaged in a sustained isometric grip task, squeezing a handheld dynamometer at 20% of their maximal voluntary contraction for 5 seconds per trial, repeated across two blocks of 20 trials/block. Real-time visual force feedback was provided to support task performance. EMG was concurrently recorded from bilateral FDI, extensor and flexor digitorum, and biceps muscles. Recording from the nonperforming (less affected) extremity enabled for the monitoring of mirror movements.

Signal preprocessing followed established procedures using EEGLAB (MATLAB 2017b, MathWorks) [49], [50]. Data were bandpass filtered (0.5–50 Hz), re-referenced to the average scalp signal, and epoched (1s pre- and 6s post-grip task onset) with temporal padding to minimize edge effects. Electrodes positioned on the cheeks and neck were excluded, retaining 194 scalp sites for analysis. Trained research staff visually inspected the EEG data and interpolated electrodes exhibiting excessive noise. Artifacts related to ocular, cardiac, and muscle activity were identified and removed via ICA (Infomax algorithm) followed by additional visual inspection.

Data were standardized so the left hemisphere corresponded to the ipsilesional hemisphere. Epochs were locked to the onset of the grip task and baseline-corrected from −500ms to −200ms in the time domain. To reduce edge artifacts during spectral analysis, all epochs were concatenated into a single continuous time series prior to wavelet convolution.

### C. Corticomuscular Coherence

CMC was computed between electrodes overlying iM1 and the FDI muscle, as the FDI plays a role in maintaining grip precision and stability [51], using a custom time-frequency spectral coherence analysis in MATLAB.

A 250 ms sliding window with 50% overlap was applied to balance temporal resolution with spectral stability, providing approximately 3 to 7 cycles of beta-band activity (13–30 Hz), which is sufficient for reliable estimation of beta oscillatory coherence while allowing sensitivity to time-varying cortico-muscular dynamics. Time-resolved signals input into CMC calculation were estimated using multitaper spectral analysis. Coherence values were calculated across 40 logarithmically spaced frequencies from 1 to 50 Hz, with wavelet cycles ranging from 3 to 7 to balance temporal and spectral precision.

CMC was quantified as the magnitude-squared coherence as follows:

(1) $$C_{xy}(f)=\frac{{|S_{xy}(f)|}^{2}}{S_{xx}(f)\cdot S_{yy}(f)}$$

where $S_{xy}(f)$ is the cross-spectral density between EEG and EMG, and $S_{xx}(f)$, $S_{yy}(f)$ are the auto-spectral densities of EEG and EMG, respectively. The beta-band CMC (13–30 Hz) was extracted for estimation of pmCMC.

### D. Phase Modulation of Corticomuscular Coherence

Computation of pmCMC was implemented in MATLAB using custom scripts. Instantaneous prefrontal delta phase was quantified by convolving a 3-cycle Morlet wavelet centered at 2 Hz with the electrical activity from electrodes overlying the prefrontal cortex (centroid at AFz). The signal was estimated over a larger window and then trimmed around the five-second grip task to reduce edge artifacts.

For each participant, the time-resolved beta-band CMC waveform was aligned with the delta-phase time series from PFC. Timepoints were binned into 30 equally spaced delta-phase bins, and the mean beta-band CMC within each bin was calculated. Coupling strength was then quantified using the mean vector length (MVL) as follows:

(2) $$pmCMC=|\frac{1}{N}\sum_{n=1}^{N}CMC_{n}\cdot e^{i\phi_{n}}|$$

where $CMC_{n}$ is the beta-band CMC at time n, and $\phi_{n}$ is the corresponding delta-phase value. This method was chosen for its robustness against inflation and artificial coupling due to non-sinusoidal components [52]. Artifacts that can arise from a non-sinusoidal waveform were minimized by the use of three spatially separated signals in the calculation of pmCMC: prefrontal delta phase, phase of the motor beta amplitude envelope, and beta phase of the high-frequency amplitude of EMG signal in the FDI.

To normalize pmCMC, surrogate data were generated by random circular shifting of the CMC time series relative to the delta-phase series by ≥ 10% of their total length [36]. This was repeated 1,000 times to create a null distribution, against which observed values were z-scored to yield a normalized pmCMC index.

To investigate the influence of PFC delta oscillatory activity on iM1–FDI beta-band CMC, we computed an exhaustive comodulogram of coupling strength between low-frequency PFC phase (1–8 Hz) and higher-frequency M1–FDI CMC amplitude (8–50 Hz) in 0.25 Hz increments. Significance was assessed using a cluster-based permutation test across participants, with clusters formed from adjacent time–frequency points exceeding a threshold of p < 0.05. Cluster mass was defined as the sum of t-values within each cluster, and permutation-based cluster correction for mass was used with a minimum cluster size of 300 to control for multiple comparisons (previously described [29]).

### E. Imaging Acquisition and Lesion-Related Injury

Structural magnetic resonance imaging (MRI) was acquired during the participants’ hospitalization using either 1.5T or 3T Siemens platforms (MAGNETOM Aera, TrioTim, or Skyra). Scanning included a high-resolution 3D T1-weighted MPRAGE sequence (1 mm^3^ isotropic voxels) and a T2-weighted FLAIR scan to enhance lesion visualization. Lesion masks were manually drawn in native space using MRIcron, with validation from a board-certified neuroradiologist. Individuals with direct lesion involvement to the PFC and/or M1 were removed from subsequent analyses.

Lesion masks were binarized and spatially transformed to Montreal Neurological Institute standard stereotaxic space. We computed overlap injury as the percentage of lesionvoxel overlap from the CST and corticostriatal tract from the Johns Hopkins University white matter tractography and Basal ganglia tracts atlases, respectively [53], [54], [55].

### F. Clinical Status and Recovery Outcomes

Clinical assessments were performed around the time of IRF admission and discharge to quantify functional status, motor impairment, and global cognition.

Functional independence was assessed using the Quality Indicators (QI) for Self-Care and Mobility from the IRF Patient Assessment Instrument. These assessments were administered by certified physical and occupational therapists. The sum of the self-care and mobility scores were computed for each time point, where higher scores indicate greater functional independence. Motor impairment was assessed using the Upper extremity Fugl-Meyer (UEFM), using both total score and subscores (proximal, wrist/hand, and coordination/speed). Global cognitive status was evaluated using the Montreal Cognitive Assessment (MoCA).

To account for individual differences in baseline impairment, a “change realized” score was calculated for each participant. This metric normalizes the raw change in behavior (Discharge–Admission) by the participants recovery potential, defined as (Maximum possible score–Admission) [56]. The realized score ranges from 0 to 1, with higher values indicating greater recovery relative to an individual’s potential for recovery.

### G. Statistical Analysis

Statistical analyses were conducted in JMP Pro 17 (SAS Institute, Cary, NC). Changes in pmCMC across IRF stay were evaluated using pairwise t-tests. Associations between pmCMC and stroke-related injury and clinical measures were assessed using Pearson or Spearman rank correlations. An initial significance threshold of *α* = 0.05 (two-tailed) was used for all inferential tests. To control for type I error across our exploratory correlation analyses, a false discovery rate (FDR) correction was applied using the Benjamini–Hochberg procedure, with P_adjusted_ < 0.05 considered statistically significant [57]. Hypothesis-driven primary comparisons, including the change in pmCMC across IRF stay, were evaluated without FDR correction.

Control analyses were conducted to assess the specificity of pmCMC effects, including analyses of prefrontal delta power, beta-band corticomuscular coherence, and direct prefrontal–EMG coupling. Effect sizes are reported where appropriate, including Cohen’s d (*d*) for pairwise comparisons.

## III. Results

### A. Participant Cohort

The enrolled cohort, consisting of 30 individuals (14 females) with stroke (10.4 ± 3.5 days post-stroke; 67.0 ± 9.8 years of age) received inpatient rehabilitation for an average of 14.0 ± 6.2 days. Of those 30 participants, 21 successfully completed the grip task with their affected extremity at around the time of IRF admission and 26 around the time of discharge. Because of direct lesion involvement to PFC and/or M1, three participants were excluded from subsequent analyses. This cohort was previously described in related but separate work [6], [58]. As summarized in Table I, participants demonstrated considerable heterogeneity with regards to stroke-related injury, baseline clinical characteristics.

### B. Activity Across the Scalp

**Fig. 1.**
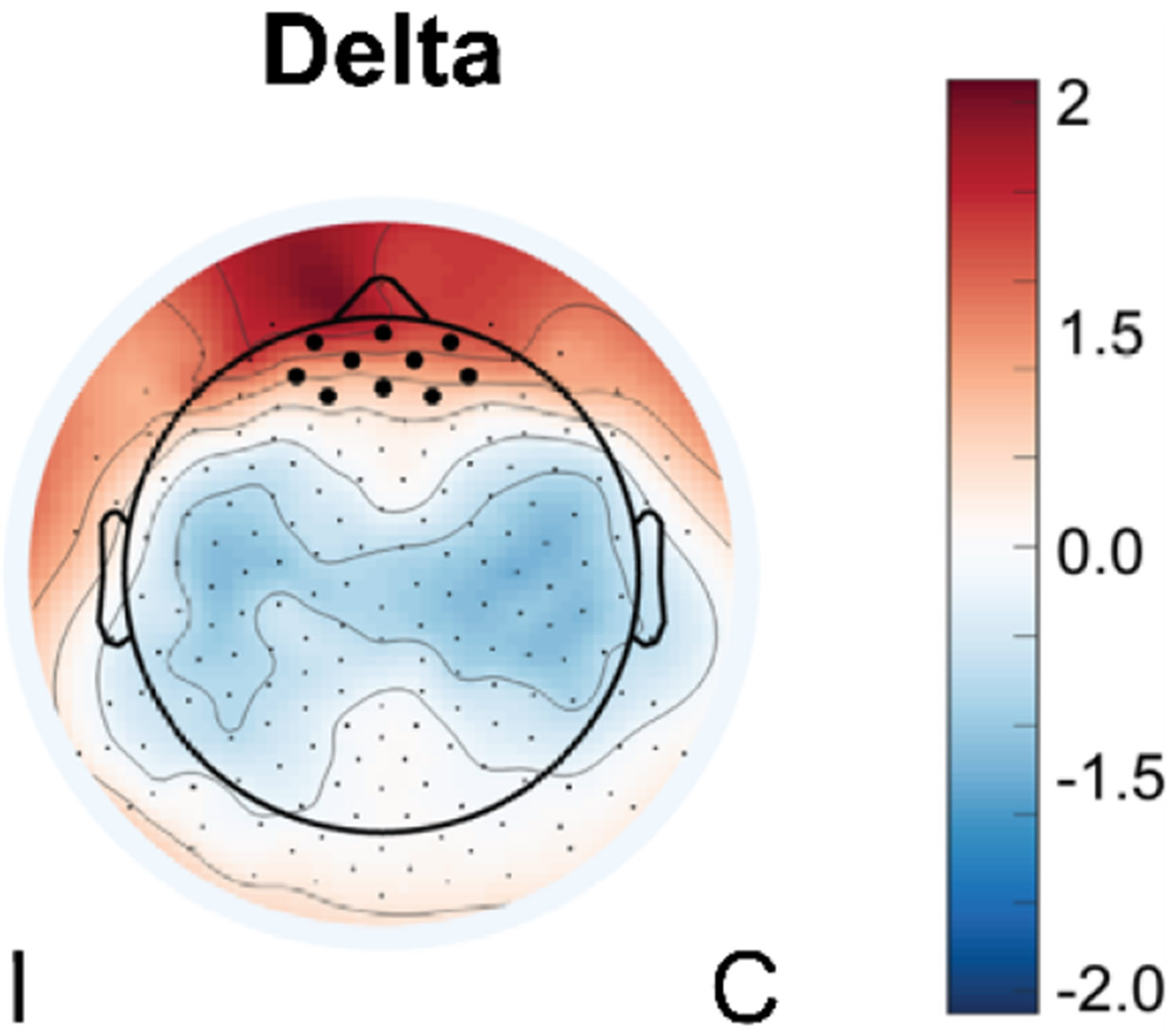
Z-transformed delta power across the scalp during the entire precision isometric grip task averaged across participants and timepoints. Electrodes overlying the anterior PFC (black electrodes) were selected for subsequent phase-modulation analyses. I, ipsilesional hemisphere; C, contralesional hemisphere.

To identify the more localized and relevant phase and amplitude sources, we visualized delta and beta oscillatory activity during task performance. This supported our choice of anterior PFC electrodes for delta-phase extraction in subsequent analysis (Fig. 1).

**Fig. 2.**
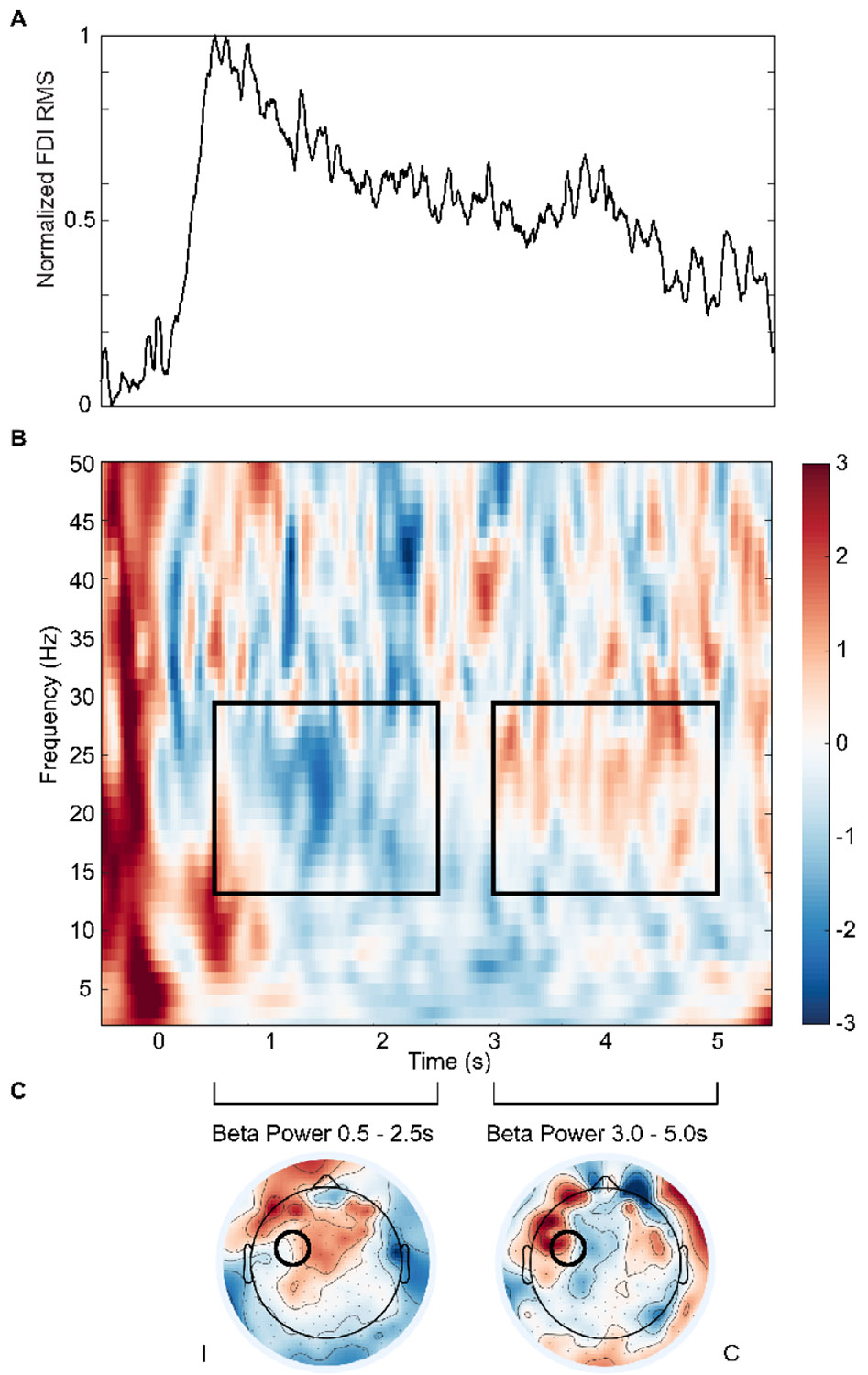
(A) Normalized FDI EMG root mean square (RMS) activity and (B) z-transformed beta-band (13–30 Hz) power from ipsilesional M1 during the precision grip task, averaged across all participants and timepoints. Black outlined regions denote the analyzed time windows: 0.5–2.5 s (early) and 3–5 s (late). (C) Scalp topographies of z-transformed beta power were averaged over these respective windows, illustrating the spatial distribution of beta activity. The location of the ipsilesional M1 electrodes used for analysis is outlined in black. The grip task spanned a time window from 0 to 5 seconds. I, ipsilesional hemisphere; C, contralesional hemisphere.

The spectrogram of iM1 activity during the task reveals dynamic modulation of beta power (Fig. 2). Specifically, beta activity transiently decreases around movement initiation (0s), which may reflect the release of inhibition required for initiating voluntary movement [59], [60] (Fig. 2B and 2C). Following this suppression, an elevation in beta power occurs between 3–5 seconds (Fig. 2B and 2C), coinciding with the sustained isometric grip phase (Fig. 2A). This temporal pattern is inversely related to the EMG RMS trace (Fig. 2A), which rises during movement initiation and stabilizes during the sustained contraction.

### C. M1-FDI Corticomuscular Coherence

**Fig. 3.**
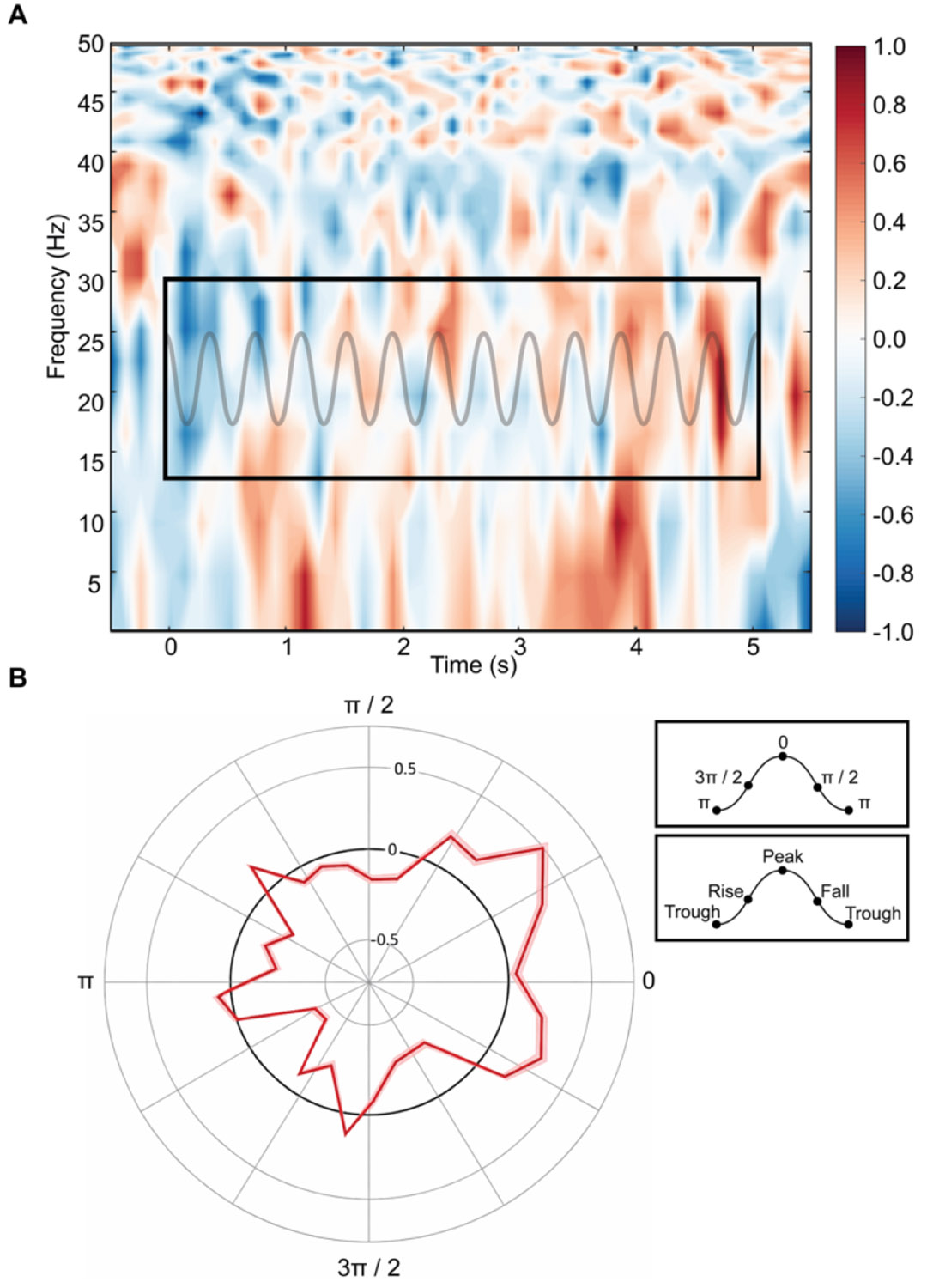
(A) Group- and time-averaged baseline-corrected CMC activity between M1 and FDI. The black box outlines the beta frequency range over the full 5-second task duration. Overlaid is a 3 Hz sine wave (faded gray) to illustrate rhythmic fluctuations within the beta-band CMC, consistent with delta-phase modulation. (B) Circular phase distribution (rose plot) depicting beta-band CMC amplitude to PFC delta phase. The red trace represents the group mean, with shaded boundaries indicating standard error. CMC magnitude exhibits a non-uniform phase preference, with maximal values occurring near the peak of the delta cycle, and reduced values near the trough. Peak and trough values in radians provide additional orientation. This temporal alignment supports the hypothesis that prefrontal delta activity dynamically modulates beta-band CMC during sustained effort.

To evaluate brain-muscle connectivity, we computed beta-range CMC between EEG electrodes overlying iM1 and surface EMG from the FDI. The time–frequency representations revealed increased coherence in the high beta range (20–30 Hz) during the active contraction period (Fig. 3). This coherence peaked approximately 2.5 seconds after task onset, in-line with the exerted EMG maintained at the target threshold (Fig. 2A) and iM1 beta power (Fig. 2B) and remained elevated during the sustained grip phase. Additionally, visual analysis confirms beta-band CMC demonstrated bursting properties roughly 3 times per second in line with the phase of delta oscillatory activity.

### D. Phase-Modulated Corticomuscular Coherence

To investigate the influence of PFC delta oscillatory activity on iM1-FDI beta-band coherence, an exhaustive comodulogram of coupling strength was run. Peak coupling strength was found in the frequency pair: 3.25–25.0 Hz and ranged from 2.25–3.75 Hz and 17.5–30.25 H, indicating frequency specificity of pmCMC from PFC to iM1-FDI between delta-phase (canonically 1–4 Hz) and beta CMC (canonically 15–30 Hz).

### E. Increases in Phase-Modulated Corticomuscular Coherence During Inpatient Rehabilitation

**Fig. 5.**
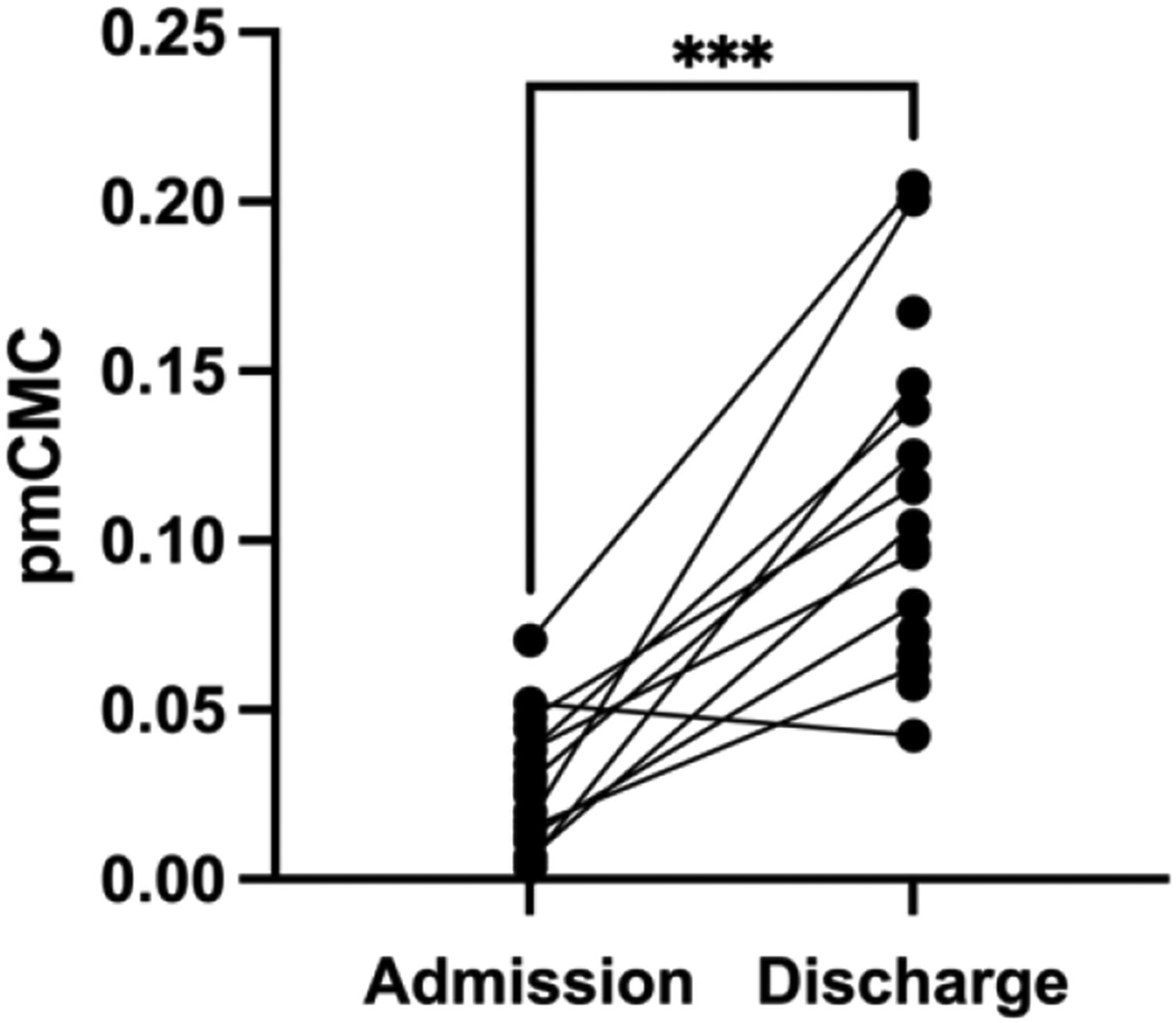
Paired t-test showing the change in pmCMC across hospitalization. *** denotes p< 0.001 (n= 18).

Overall, participants spent around 2 weeks in the IRF (Table I). During this time, pmCMC significantly increased between IRF admission and discharge timepoints (*t*_(18)_ = 7.13, *P* < 0.001, *d* = 1.84, Fig. 5).

### F. Phase-Modulated Corticomuscular Coherence Relates to Corticostriatal Injury

**Fig. 6.**
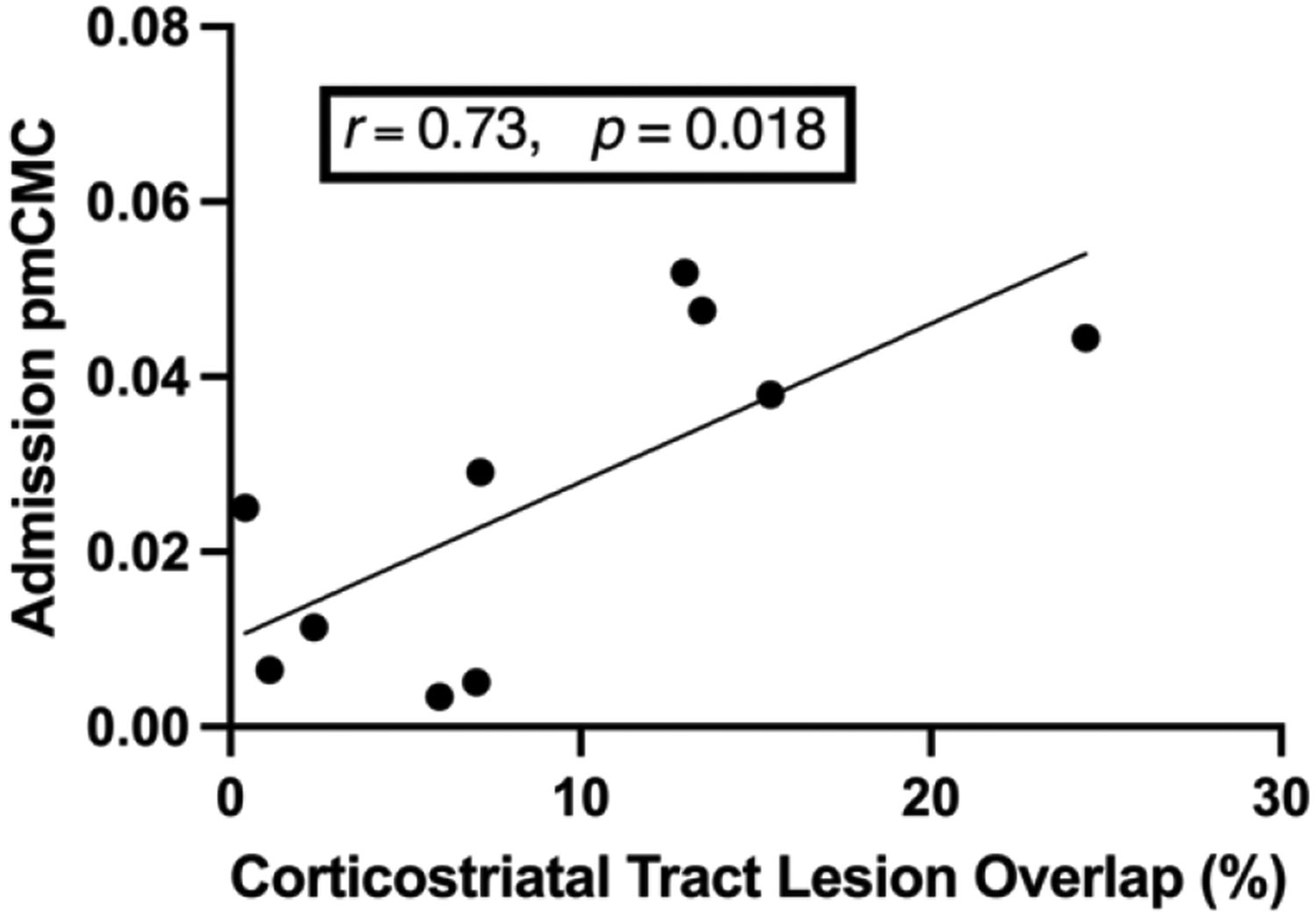
Correlation between pmCMC at the time of admission with percent lesion overlap injury with the corticostriatal tract (n= 10).

To assess whether pmCMC reflects compensatory responses to lesion-related injury, we determined relationships between pmCMC with corticostriatal and CST lesion overlap injury. At admission, in a subsample of 10 participants with direct lesion damage to the corticostriatal tract, pmCMC significantly correlated with the magnitude of lesion overlap (*r* = 0.73, *P* = 0.018, *P_adjusted_* < 0.050, Fig. 6). In a subsample of 17 participants with damage to the CST, CST overlap injury did not correlate with pmCMC (*ρ* = 0.20, *P* = 0.468, *P_adjusted_* > 0.050).

### G. Phase-Modulated Corticomuscular Coherence Aligns With Motor Coordination and Functional Independence

**Fig. 7.**
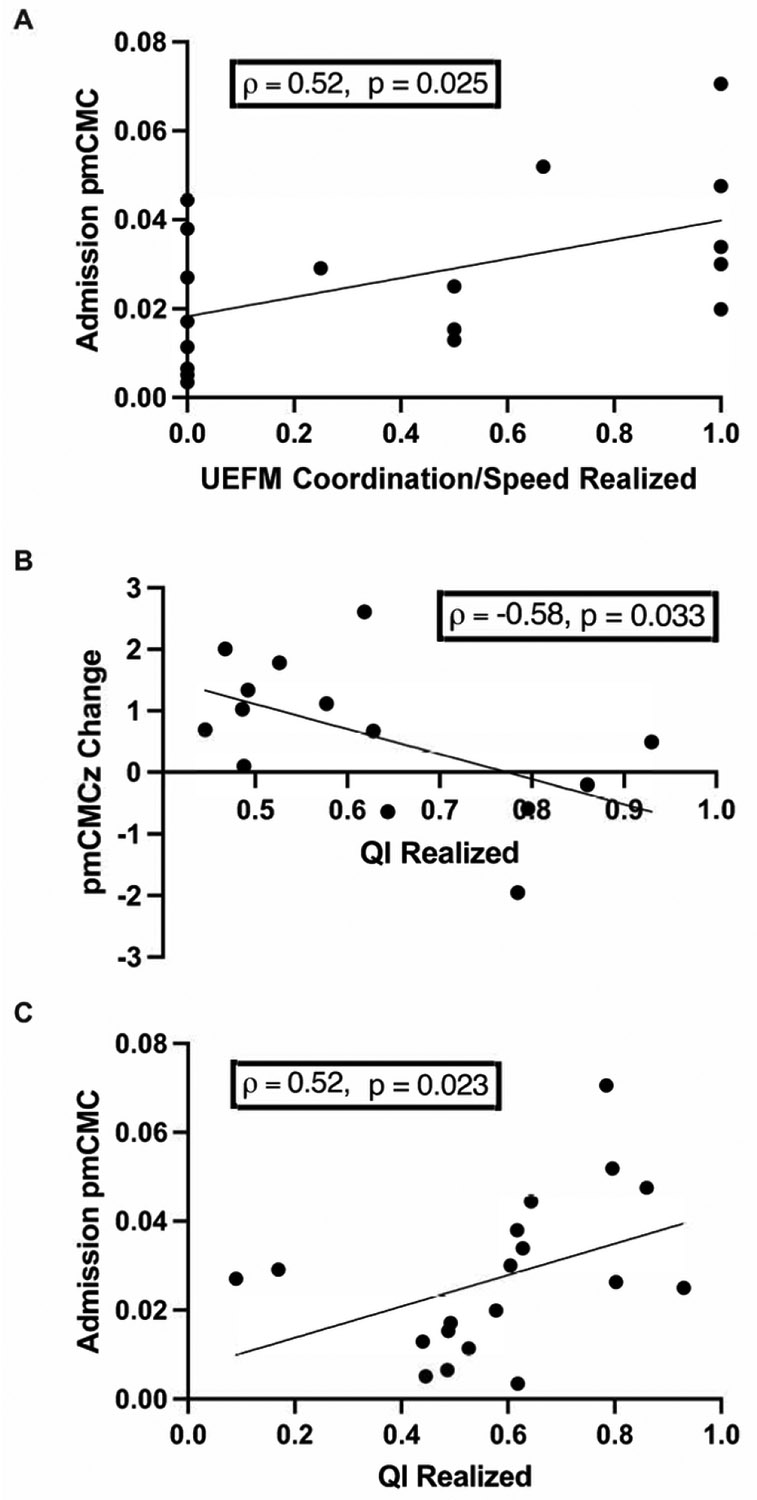
Correlations between (A) pmCMC at admission with the realized change of the UEFM Coordination /Speed subscore (n= 19) (B) the change in pmCMCz and realized recovery functional independence (n= 14), and (C) pmCMC at admission and the realized recovery in functional independence (n= 19).

Correlation analyses revealed that pmCMC around the time of IRF admission did not correlate with UEFM scores at admission or discharge, nor did the change in pmCMC correlate with the realized change in UEFM (*ρ* = 0.05–0.35, *P* > 0.050). Examination of pmCMC relationships with UEFM subscores yielded a positive relationship between pmCMC at admission and the realized recovery of coordination/speed (*ρ* = 0.52, *P* = 0.025, *P_adjusted_* < 0.050, Fig. 7A).

Similar analyses were performed to evaluate the association between pmCMC and functional independence. Greater pmCMC at admission related to greater gains in functional independence (*ρ* = 0.52, *P* = 0.023, *P_adjusted_* < 0.050, Fig. 7C). In contrast, greater *change* in pmCMC during hospitalization related to smaller gains in functional independence (*ρ* = −0.58, *P* = 0.033, *P_adjusted_* < 0.050, Fig. 7B).

### H. Phase-Modulated Corticomuscular Coherence Relates to Cognition

**Fig. 8.**
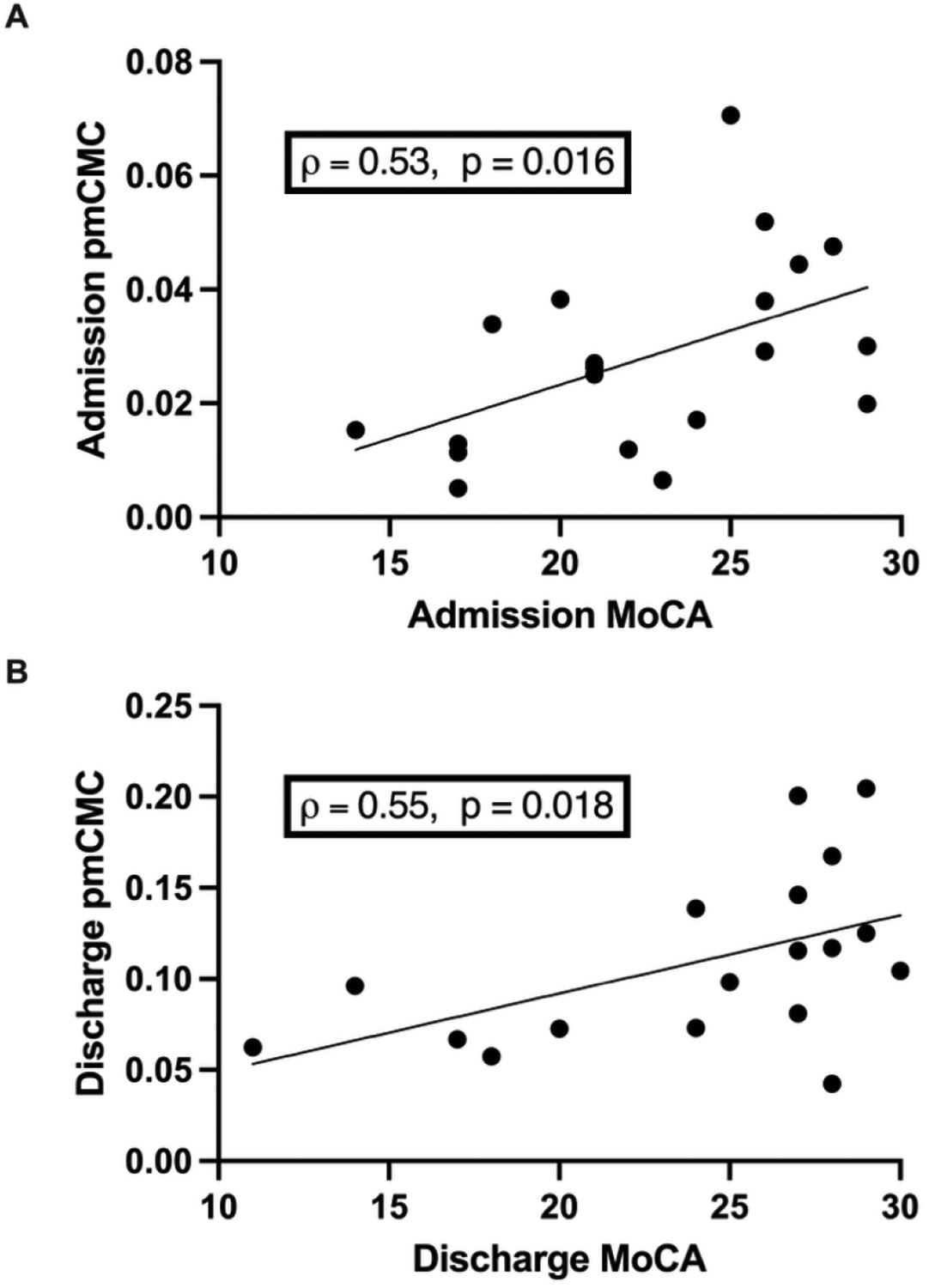
Correlations between pmCMC and MOCA scores at the time of (A) admission, and (B) discharge. (n= 19).

Multivariate correlations were also performed to determine relationships between pmCMC and cognitive function. Crosssectional analyses showed that greater pmCMC was associated with less cognitive impairment at both the time of admission (*ρ* = 0.53, *P* = 0.016, *P_adjusted_* < 0.050, Fig. 8A), and discharge (*ρ* = 0.55, *P* = 0.018, *P_adjusted_* < 0.050, Fig. 8B). Additionally, pmCMC at admission positively correlated with the change in cognition during hospitalization (*ρ* = 0.55, *P* = 0.014 *P_adjusted_* < 0.050).

### I. Control Analyses

The above analyses were repeated for both PFC delta power and beta-band iM1-FDI CMC to assess the specificity of pmCMC to stroke recovery. Corticostriatal lesion load did not correlate with either PFC delta power (*r* = −0.09, *P* = 0.818) or CMC (*r* = 0.49, *P* = 0.124). Similarly, there was no difference in PFC delta power across hospitalization (*t*_(18)_ = 0.56, *P* = 0.586, *d* = −0.14). Beta M1-FDI CMC demonstrated an increase across hospitalization (*t*_(18)_ = 2.26, *P* = 0.043, *d* = 0.63).

Baseline PFC delta power significantly correlated with UEFM realized change (*ρ* = 0.53, *P* = 0.025); however, this relationship did not survive FDR correction (*P_adjusted_* > 0.05). No associations were observed between PFC delta power and the UEFM coordination/speed subscore, MoCA, or QI scores at admission, discharge, or change across hospitalization.

CMC at admission did not correlate with any behavioral measures (*P* > 0.05). Greater CMC around the time IRF discharge related to less realized recovery (UEFM total: *ρ* = −0.47, *P* = 0.050; UEFM coordination/speed subscore: *ρ* = −0.47, *P* = 0.049). These associations did not persist following FDR correction procedures (*P_adjusted_* > 0.05).

To ensure these results were specific to EEG-CMC coupling, an additional control analysis was performed where coupling was estimate between PFC delta phase and FDI beta amplitude. No change in direct PFC-FDI coupling occurred across hospitalization (*t*_(18)_ = 0.83, *P* = 0.416, *d* = 0.23) nor did PFC-FDI coupling correlate with motor or cognitive function (*ρ* = −0.32 – 0.24, *P* > 0.05) at admission. We observed a significant association between PFC-FDI coupling measured at discharge with MoCA score also obtained at discharge (*ρ* = 0.47, *P* = 0.023), though, this association did not survive FDR correction (*P_adjusted_* = 0.052).

## IV. Discussion

In this study, we introduce pmCMC as a novel biomarker of cognitive-motor integration after stroke. Our findings support a model where stroke recovery is not merely a restoration of isolated motor pathways but requires dynamic reorganization of large-scale brain networks that link cognitive, sensorimotor, and peripheral muscular systems [1], [61]. By combining cross-frequency coupling with brain-muscle connectivity, pmCMC offers a window into how prefrontal executive control influences peripheral motor execution in real time and how these interactions characterize both stroke-related injury and recovery.

As a foundation, we first examined scalp-level oscillatory power to verify the physiological relevance of our chosen frequency bands and anatomical regions. Delta power was most prominent over anterior frontal electrodes, consistent with the PFC’s known role in executive and attentional control [35]. By comparison, beta-band power was localized over iM1 and bilateral parietal regions during the isometric grip task, aligning with prior work implicating these regions in motor preparation and sustained isometric effort [62]. During task performance, iM1 beta activity exhibited a phasic trajectory, briefly decreasing at movement onset and then rebounding and sustaining during the grip phase, consistent with the temporal dynamics of force production during the rising (recruitment) and/or holding (sustainment) components of the grip task [63]. Next, we evaluated CMC between M1 and the FDI muscle. As expected, CMC peaked in the upper beta range (20–30 Hz) during the sustained contraction period and displayed rhythmic bursts that were aligned with the phase of delta activity. These findings confirm that our task engaged canonical motor control networks and provided a foundation from which to probe more integrative measures of motor-cognitive coupling.

Our preliminary findings support the view of stroke recovery as an inherently network-level process, one that engages interacting, large-scale brain networks rather than isolated regions or linear pathways [64]. Following neural injury, individuals engage compensatory mechanisms that involve not only M1 but also distributed networks including the PFC, parietal regions, and subcortical structures [64], [65]. This distributed plasticity, often involving cognitive scaffolding, may arise in response to diaschisis-related effects after stroke, where a focal lesion produces widespread network disruption [66]. Despite evidence emphasizing the global nature of post-stroke neural disruption and plasticity, most electrophysiological biomarkers of stroke recovery continue to focus narrowly on either motor [67] or cognitive domains in isolation [68]. Well-established measures such as beta-band CMC capture efferent motor drive from M1 to the periphery but neglect how these signals are modulated by cognitive demands [47], [69]. Conversely, prefrontomotor PAC may index cognitive control over the motor cortex but does not directly quantify its impact on motor execution [6], [36]. Based on our findings, we posit that pmCMC serves as a more integrative marker of recovery as it quantifies the dynamic phase-dependent modulation of motor beta CMC by prefrontal delta activity.

We hypothesized that the trajectory of pmCMC would mirror that of delta-beta prefrontomotor PAC based on prior work showing initially elevated PAC relative to controls during early post-stroke recovery, that decreased over time [6]. We attributed reductions in PAC to a decreased reliance on cognitive contributions to motor system function. Here, we observed an increase in pmCMC during inpatient hospitalization, which may infer key distinctions between PAC and pmCMC measurement with the former indexing the coordination two spatially distinct cortical signals and the latter representing the extent to which that coordination influences brain-muscle functional coupling. Hence, pmCMC conveys cognitive-motor integration at the level of motor output coupling.

Of note, the trajectory of pmCMC resembled that of beta-band CMC observed during early post-stroke recovery where increases in CMC paralleled sensorimotor pathway reorganization [70], [71]. Applied to this work, the increase in pmCMC may signify a reorganization of motor control mechanisms wherein prefrontal systems increasingly coordinate motor output as sensorimotor function improves. This recognizes the role of PFC in both executive function and premotor processes [72], [73], including motor inhibition and response selection [74], [75], that are often disrupted following stroke [76]. Refinement of motor output from PFC likely involves both excitatory and inhibitory pathways [77], [78].

Supporting the relevance of these pathways, greater pmCMC positively related to lesion overlap injury involving the basal ganglia-corticostriatal network but not corticospinal. This pattern suggests that pmCMC may be particularly sensitive to disruptions in frontostriatal circuitry–regions involved in integrating cognitive control with motor planning and initiation [43], [79]. The basal ganglia supports the gating and modulation of motor activity via communication with PFC, and damage to these systems likely necessitates the deployment of executive control strategies [80] and, hence, upreglation of pmCMC. However, it is important to note that the extent of corticostriatal injury in our sample was modest, with lesion overlap reaching a maximum of approximately 22%. Thus, the observed associations may reflect relatively subtle variations in corticostriatal engagement, rather than widespread structural damage. The lack of significance between CST overlap injury and pmCMC underscores the importance of basal ganglia-corticostriatal integrity in supporting the neural dynamics indexed by pmCMC.

In addition to injury, we also observed significant associations between pmCMC and assessments of motor coordination/speed, cognition, and functional independence. This suggests that the neural dynamics captured by pmCMC, likely representing the integration of cognitive and motor system function, are particularly salient in these performance and functional measures. Importantly, we found that greater pmCMC at admission was associated with greater gains in functional independence during hospitalization, whereas increases in pmCMC over time were inversely related to those gains. Together, these results suggest that pmCMC is not a monotonic biomarker of recovery but, instead, signifies the dynamic engagement of hierarchical cognitive-motor control mechanisms that differentially support compensatory or restorative processes depending on recovery stage. Higher pmCMC early after stroke may reflect reorganization and/or compensation involving the recruitment of prefrontal control mechanisms that facilitate recovery potential, whereas increases in pmCMC over time during may signify continued reliance on cognitive scaffolding rather than the restoration of efficient motor execution.

Control analyses confirmed that the observed pmCMC effects were not driven by changes in delta power or beta-band CMC. Although frontal delta oscillations have been linked to arousal and vigilance [81], [82], converging evidence indicates that prefrontal delta activity during sustained goal-directed tasks denotes executive engagement and top-down control [36], [83]. In our data, the absence of associations between delta power and behavioral or injury measures, combined with the specificity of the phase-dependent pmCMC effects, argue against an arousal-based explanation. Instead, the phase-dependent modulation captured by pmCMC supports its role as a distinct marker of cognitive control over motor execution. Moreover, the lack of robust PFC-EMG coupling effects underscores the importance of modeling M1-muscle coherence as an intermediary, consistent with the view that cognitive control influences motor output primarily by modulation of corticospinal communication rather than through direct prefrontal-peripheral coupling. This specificity highlights pmCMC’s potential clinical utility for identifying when and how executive systems operate when for impaired motor pathways.

From a methodological perspective, pmCMC advances beyond conventional connectivity analyses by integrating elements of both CMC and cross-frequency coupling (PAC). As Cohen notes in *Analyzing Neural Time Series Data*, most connectivity measures in cognitive electrophysiology are bivariate because they are interpretable, computationally simplistic, and supported established statistical frameworks [84]. Yet, such a pragmatic approach may oversimplify complex brain dynamics, particularly in systems characterized by multivariate interactions [84]. Emerging modeling frameworks in neuroscience emphasize the *form* of interaction among brain regions rather than just its presence [85], [86].

Our approach acknowledges this limitation while adopting a hybrid strategy. Although pmCMC is formally bivariate, it incorporates three critical signals: prefrontal delta phase, M1-muscle beta coherence, and their cross-frequency modulation within a single analytic framework. This allows pmCMC to capture hierarchical, task-relevant cognitive-motor interactions while retaining the interpretability and statistical strengths of bivariate measures. In this way, pmCMC bridges classical coherence analyses and emerging network-level models, maintaining practical applicability while fostering circuit-informed interpretations.

Several limitations warrant consideration. The modest sample size necessitates cautious interpretation and generalization across stroke. Relatedly, to avoid model overfitting due to insufficient statistical power and potential bias given the heterogeneity of our cohort, covariate adjustment was not performed. This includes covariates relevant to cognitive and motor function such as age, education, lesion volume, and length of IRF stay. Future, larger-scale studies are needed to examine both covariate-adjusted and unadjusted effects. We Importantly, pmCMC does not imply causality between prefrontal delta activity and beta-band CMC. Rather, pmCMC reflects a temporal relationship where corticomuscular coupling is preferentially expressed at specific phases of prefrontal delta oscillations. Although time-resolved beta-band CMC exhibited transient fluctuations during task performance, these fluctuations should not be interpreted as discrete bursts that are rigidly aligned to specific phases of prefrontal delta activity. Measurement of pmCMC does not rely on identifying individual coherence bursts but instead quantifies whether the magnitude of corticomuscular coupling is statistically organized by prefrontal phase across time. As such, apparent temporal alignment should be interpreted as evidence of structured coordination rather than precise phase locking or causal entrainment. Given the limitations of causal inference with pmCMC in this work, future experimental studies incorporating non-invasive brain stimulation or network perturbation would help clarify the directionality of the cognitive-motor interactions conveyed by pmCMC. Finally, extending pmCMC measurement from a unilateral upper-extremity grip task to other domains of sensorimotor control such as gait, bimanual coordination, and balance, and across a wider recovery window will are key for clinical translation.

## V. Conclusion

This study introduces pmCMC as a novel biomarker of cognitive-motor integration during stroke recovery. By capturing the real-time modulation of motor output by prefrontal executive control, pmCMC provides unique insights into compensatory mechanisms underlying functional improvement. This work contributes to a growing movement toward integrative neurorehabilitation models, where cognition and motor control are understood as dynamically intertwined processes. Future studies should expand upon this framework to develop targeted interventions that leverage cognitive-motor coupling for optimized recovery trajectories.

## Figures and Tables

**Fig. 4.**
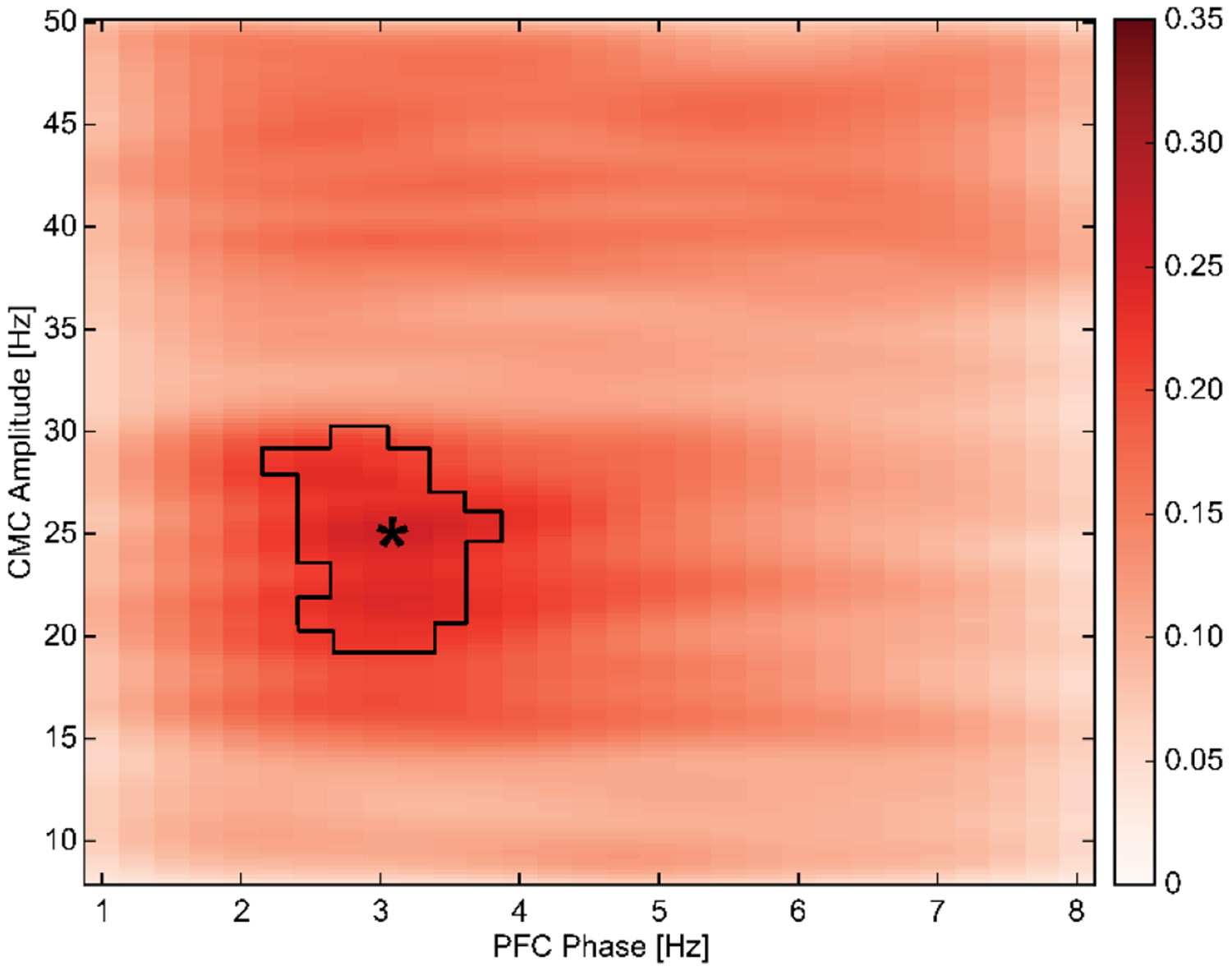
Comodulogram illustrating the group average pmCMC between prefrontal delta phase (1–8 Hz) and iM1–FDI CMC amplitude (8–50 Hz). Outline was significant at P < 0.05 and permutation-based cluster correction, highlighting peak modulation. Peak CMC is at the pair: 3.25–25.0 Hz.

**TABLE I:** Participant Demographics

Descriptor	n	Mean (SD) or Median [IQR]	Range
Sex			
Female	14	–	–
Male	16	–	–
Race			
Black	6	–	–
White	24	–	–
Age (years)		67.0 (9.8)	51–85
Lesioned Hemisphere			
Left	12	–	–
Right	18	–	–
Stroke Type			
Hemorrhagic	2	–	–
Ischemic	28	–	–
Dominant Hemisphere Injury			
Yes	10	–	–
No	20	–	–
Handedness Prior to Stroke			
Left	1	–	–
Right	29	–	–
Days in IRF		15.5(6.3)	4–29
Lesion Volume (cc)		19.9 (35.9)	0.08–155.7
CST Overlap (%)	29	8.9 (6.6)	0.02–22.2
UEFM (max=66)			
Visit 1		40.9 (25.1)	2–66
Visit 2	29	46.8 (23.6)	4–66
Change	29	6.4 (9.6)	−7–41
MoCA (max=30)			
Visit 1	28	22.3 (4.2)	14–29
Visit 2	29	23.9 (5.4)	11–30
Change	28	1.6 (3.2)	−7–6
Quality Indicators (max=144)			
Visit 1		53.9 (20.0)	19–87
Visit 2		98.4 (26.6)	33–140
Change		44.5 (18.1)	11–80
NIHSS (max=42)			
Visit 1		4.2 (4.1)	0–17
Visit 2	29	2.9 (3.6)	0–13
Change	29	−1.5 (2.0)	−7–2

Values presented as mean (Standard Deviation, SD) or median [interquartile range, IQR]. IRF, inpatient rehabilitation facility; NIH, National Institutes of Health Stroke Scale; cc, cubic centimeters; CST, corticospinal tract; UEFM, Upper Extremity Fugl-Meyer; MoCA, Montreal Cognitive Assessment; NIHSS; NIH Stroke Scale. Number of participants is n=30 unless otherwise noted.

## Data Availability

Data and code to support these findings are available in the UNC Dataverse repository at https://doi.org/10.15139/S3/2MFWKR.
